# Steroid precursors, steroids, neuroactive steroids, and neurosteroids concentrations in serum and saliva of healthy neonatal heifer Holstein calves

**DOI:** 10.1111/jvim.15957

**Published:** 2020-11-17

**Authors:** Monica Aleman, Munashe Chigerwe, Anita Varga, John E. Madigan

**Affiliations:** ^1^ Department of Medicine and Epidemiology School of Veterinary Medicine, University of California Davis California USA

**Keywords:** brain, cattle, consciousness, encephalopathy, neurosteroids

## Abstract

**Background:**

Persistence of high neurosteroid concentrations in blood is associated with neonatal encephalopathy and septicemia in foals. This has not been investigated in calves.

**Objectives:**

To determine concentrations of steroid compounds in serum and saliva within the first 48 hours after birth in healthy neonatal calves, identify potential markers for disease, and investigate the association between serum steroid compounds concentrations in calves and their respective dams within 2 hours after birth.

**Animals:**

Twelve healthy neonatal heifer Holstein calves and their dams.

**Methods:**

Prospective study. Serum and saliva were collected from calves at 2, 6, 24, and 48 hours after birth. Steroid compounds were analyzed using liquid chromatography‐mass spectrometry. A nonlinear regression model was used to determine half‐lives of the neurosteroids. Serum concentrations of neurosteroids between the cows and calves were compared using the Wilcoxon signed rank test.

**Results:**

Half‐lives (95% confidence intervals) of dehydroepiandrosterone (DHEA) and 17α,20α‐dihydroxyprogesterone in calf serum were 2.9 (2.1, 4.3), and 2.1 (1.3, 3.0) hours, respectively. Pregnanediol in saliva had a half‐life (95% confidence interval) of 24.5 (14.2, 66.5) hours. Serum DHEA (1718.7 ± 2313 vs 57.7 ± 44) and 17α,20α‐dihydroxyprogesterone (207.8 ± 198.2 vs 43.5 ± 33.5) concentrations respectively were higher (*P* < .05) in calves compared to cows.

**Conclusions and Clinical Importance:**

Dehydroepiandrosterone, 17α,20α‐dihydroxyprogesterone, and pregnanediol could be potential markers of disease in neonatal heifer calves with unexplained failure to thrive or encephalopathy. However, because of the wide 95% confidence interval of the half‐life, pregnanediol in saliva might not be a potential marker.

**Abbreviations**
DHEA: dehydroepiandrosterone
DHEA‐S: dehydroepiandrosterone sulfate
NAS: neuroactive steroids
NE: neonatal encephalopathy
NMS: neonatal maladjustment syndrome
NS: neurosteroids
WCS: weak calf syndrome

## 1 INTRODUCTION

Neurosteroids (NS) and neuroactive steroids (NAS) are steroid compounds produced in the brain and other tissues, respectively, both of which have effects in the nervous system.^1^, ^2^ These compounds are synthesized from cholesterol which is converted into pregnenolone and then into all other endogenous steroids.^3^ These compounds modulate states of arousal, cognitive function, mood changes, stress response, central nervous disorders, seizures, and play an important role in brain development and neuroprotection.^1^, ^3^, ^4^, ^5^, ^6^, ^7^, ^8^ Neurosteroids and NAS concentrations at birth have been investigated in infants and neonatal foals.^9^, ^10^, ^11^ Neonatal maladjustment syndrome (NMS) is the most common neurologic disorder affecting foals within the first 72 hours of life, and is also known as neonatal encephalopathy (NE), hypoxic‐ischemic encephalopathy, perinatal asphyxia, and dummy foal syndrome.^12^, ^13^, ^14^, ^15^, ^16^ This syndrome encompasses foals suffering from perinatal hypoxia, and those suspected of having impaired transition from intra‐ to extrauterine life as the result of persistent increase of NS and NAS concentrations after birth.^11^, ^17^, ^18^ This persistent increase in steroid compounds is presumed to cause alterations in behavior including lack of bonding with the mare and nursing, states of consciousness, and in some foals causing seizures.^19^, ^20^ Furthermore, IV infusion of a neurosteroid (allopregnanolone) into healthy neonatal foals produced alterations in behavior similar to those observed in NE.^21^, ^22^

A similar syndrome occurs in neonatal calves termed NE.^23^, ^24^ Other terminology includes dummy calf and weak calf syndrome (WCS) to describe neonatal calves with weakness and failure to thrive of undetermined etiology.^25^ Other etiologies of WCS include infectious and nutritional causes.^26^ A recent study of 200 neonatal calves classified 29% as NE and identified being male, dystocia, prolonged labor, and calf malpresentation as risk factors for the development of NE.^23^ If the pathophysiology of WCS is similar to that of NMS in neonatal foals, the role of neurosteroid compounds in the development of NE in neonatal calves warrants investigation.^17^, ^18^

The majority of studies evaluating concentrations of NS have analyzed serum or plasma samples.^9^, ^11^, ^18^ A potential alternative to blood include saliva and urine as in other species.^27^ However, comparison of concentrations of several NS in serum and saliva have not been investigated in veterinary medicine, especially during times of large shifts in concentrations within the first 48 hours after birth.^28^ There are mixed results in correlating serum and salivary cortisol concentrations in horses.^28^ Neurosteroids and NAS concentrations have not been investigated in neonatal calves to determine if similar shifts in concentrations occur during the first 48 hours of life similar to other species.^11^ The calf is an ideal species to investigate such a process as they have a large average birth weight allowing safe collection of multiple blood collections. Furthermore, they secrete large amounts of saliva making it easier to collect and monitor. Therefore, the objective of the study was to determine concentrations of a panel of NS and NAS in both, serum and saliva within the first 48 hours of life in healthy neonatal calves, and identify those with a rapid decline in concentrations during this time to serve as potential markers of neonatal disease. A secondary objective in this study was to investigate the association between serum steroid compounds concentrations in calves and their respective dams within 2 hours after birth.

## 2 MATERIALS AND METHODS

### 2.1 Animals

After approval by the University of California‐Davis Institutional Animal Care and Use Committee (UCD IACU #18288), Holstein heifer calves born at the University of California‐Davis Animal Science Dairy Barn were enrolled for use in this study. During the study period, bull calves from the farm of study were sold at 24 to 48 hours of age, thus were not available for use in the study. The inclusion criteria consisted of calves born of a normal gestational period (279‐287 days), birth, and physical and neurological examination. Additionally, successful nursing of colostrum by the calf within 6 hours after birth was required for enrollment in the study. The dams of the enrolled calves were considered healthy based on clinical examination.

#### 2.1.1 Sample collection and handling

After a physical and neurological examination at 2 hours after birth, blood from the jugular vein and saliva samples were collected at 2, 6, 24, and 48 hours after birth. For each calf and cow, venipuncture was performed on the left or right jugular vein. At each time point, a venous blood sample (3 mL) was collected with a 20‐gauge needle and aliquoted into a glass vacuum blood tube with no anticoagulant (Monojet red top, Pulmolab, Northridge, California). The blood was allowed to clot at room temperature for 1 hour and blood samples were then centrifuged for 10 minutes at 1000*g*. Serum was aliquoted into 2 mL cryotubes (Thermo Fisher Scientific, West Sacramento, California) and immediately stored at −80°C until analysis.

At each time point, saliva was collected from each of the calves using a salivette and dual chambered centrifuge tubes (Salimetrics LLC, State College, Pennsylvania) and dual chambered centrifuge tubes to allow for easy extraction of the saliva. The calves were allowed to suckle on the absorbent swab for 2 minutes, and then the swabs were placed within 10 seconds on ice in the provided tube. The tubes were then centrifuged for 10 minutes at 1000*g*, and the saliva was collected and frozen at −80°C until further analysis.

#### 2.1.2 Sample analysis

Serum and saliva samples were analyzed for concentrations of steroid precursors, steroids, NAS, and NS including cholesterol, cholesterol‐3‐SO_4_, pregnanolone, pregnenolone, pregnenolone‐SO_4_, pregnanediol, 17‐hydroxypregnenolone, 21‐hydroxypregnanolone, 20‐hydroxypregnenolone, epiallopregnanolone‐SO_4_, 20α‐hydroxy‐5a‐pregnan‐3‐1, 11α‐hydroxy‐4‐pregnene‐3, 11β‐hydroxy‐4‐pregnene‐3, allopregnanolone, dehydroepiandrosterone (DHEA), dehydroepiandrosterone‐SO_4_ (DHEA‐S), progesterone, 17α‐hydroxyprogesterone, 5α‐dihydroprogesterone, 20α‐dihydroprogesterone, 17α,20α‐dihydroprogesterone, 5β‐dihydroprogesterone, cortexolone, cortexone, cortisol, corticosterone, deoxycorticosterone, aldosterone, androstenedione, testosterone, allodihydrotestosterone, epitestosterone, estrone, 17‐estradiol, estradiol 3‐SO_4_, estrone‐SO_4_, estriol, estriol‐3‐SO_4_, and etiocholanolone‐3 using liquid chromatography‐mass spectrometry with online sample extraction by turbulent flow chromatography and detection by standard reference material on a triple quadrupole mass spectrometer as described.^11^

#### 2.1.3 Statistical analysis

All statistical analyses was performed using GraphPad Prism (GraphPad version 8, San Diego, California) and JMP Pro (JMP Pro version 14, SAS Institute, Cary, North Carolina). Data points distribution was tested for normality using the Shapiro‐Wilk test. Mean ± SD was reported when data were normally distributed whereas median (range) was reported when data were not normally distributed. Changes in concentrations of the NS and NAS over the first 48 hours were initially investigated to determine if their decay followed an exponential, second‐ or third‐order polynomial models. Choice of which model to use for final representation for each compound was based on the coefficient of determination (*R* ^2^). Compounds that followed a 1‐phase exponential decay model were considered preferable targets for use as reference compounds for neonatal calves. The half‐life (95% confidence interval [CI]) of these chosen compounds was calculated using nonlinear regression with random effects for initial concentration of each compound at the first blood collection (at 2 hours). In contrast, compounds after a decay that fitted best with a second or third polynomial model were considered poor targets for use as reference compounds in neonatal calves. When a compound was considered a potential reference target in both serum and saliva, rate constants were used to compare differences in rate of decay between the nonlinear regression models using an *F* test. Concentrations of NS between the paired calves and cows at 2 hours were compared using a Wilcoxon signed rank test. Significance was assigned at *P* < .05.

## 3 RESULTS

Ten cows and 12 neonatal heifer calves (2 pairs of twins) were included in the study. Cows age ranged from 3 to 4 years old. Median (range) concentrations for neonatal calf steroid precursors, steroids, NAS, and NS in serum and saliva are shown in Table 1.

**TABLE 1:** Mean ± SD serum and saliva steroid compounds concentrations (pg/mL) at each time point in dairy calves from birth to 48 hours of life (N = 12). * represents steroid compounds undetectable in serum or saliva

	Birth (2 hours)		6 hours		24 hours		48 hours	
Steroid compound	Serum	Saliva	Serum	Saliva	Serum	Saliva	Serum	Saliva
Aldosterone	374.9 ± 1213	*	222.7 ± 567.8	*	402 ± 689.8	*	551.3 ± 792.5	*
Allodihydrotestosterone	64.4 ± 45.8	179.5 ± 371.3	111.6 ± 155.2	174.7 ± 213.2	70.6 ± 38.7	174.3 ± 212.6	45.8 ± 43.5	80.6 ± 94.8
Allopregnanolone	0	0	5.0 ± 17.3	0	92.8 ± 100.6	0	157.4 ± 112.8	35.3 ± 111.6
Androstenedione	2 ± 5.4	44.4 ± 51.9	0.6 ± 2	452.4 ± 1078	1.9 ± 5.7	179.2 ± 205.6	4.0 ± 7.8	177.3 ± 144.4
Androstenolone (DHEA)	1718.7 ± 2313	1875 ± 2715	873.3 ± 736.3	3147 ± 4730	291.2 ± 461.7	11 112 ± 18 943	265.9 ± 249	5182 ± 5791
Cholesterol‐3‐SO_4_	*	2 053 664 ± 2 754 563	*	2 017 214 ± 1 696 807	*	981 887 ± 752 266	*	601 802 ± 308 121
Corticosterone	624.1 ± 562.6	1049 ± 950.6	387.8 ± 290.1	3548 ± 4450	271.1 ± 222.3	2136 ± 1905	209.6 ± 170.2	1361 ± 1316
Cortexone	76.7 ± 46.3	214.1 ± 522.4	41 ± 35.2	206.4 ± 229.8	20.3 ± 21.4	237.5 ± 215.3	16.9 ± 14.3	141.0 ± 146.2
Cortexolone	3.6 ± 11.3	107.1 ± 164.9	21.7 ± 52.2	312.0 ± 594.5	6.1 ± 13.5	152.2 ± 249.3	9.6 ± 18.5	36.8 ± 113.8
Cortisol	35 611 ± 39 866	4403 ± 5857	31 598 ± 30 250	1957 ± 3122	17 764 ± 15 177	230.5 ± 443.8	13 953 ± 9773	0
Dehydroepiandrosterone‐SO_4_ (DHEA‐S)	*	413.6 ± 322.9	*	557.2 ± 653.8	*	320.9 ± 375.4	*	249.1 ± 170.7
5α‐Dihydroprogesterone	16.8 ± 49.2	88 ± 178.1	13.4 ± 31.4	319.7 ± 794.7	8.6 ± 20.3	288.2 ± 931.7	5.6 ± 13.3	1024 ± 2712
5β‐Dihydroprogesterone	68.6 ± 62.2		33.8 ± 46.3		13.6 ± 18.4		90.8 ± 135.2	
20α‐Dihydroprogesterone	266.8 ± 244.1	7.7 ± 17.1	74.9 ± 81.8	1.4 ± 4.8	26.6 ± 31.5	35.2 ± 48.3	36.7 ± 31.1	21.7 ± 29
17α,20α‐Dihydroxyprogesterone	207.8 ± 198.2	103.5 ± 118.8	66.2 ± 74	106.7 ± 138.2	17.4 ± 21.6	26.2 ± 48.4	14.7 ± 15.5	43.0 ± 44.9
Epiallopregnanolone‐SO_4_	*	17.6 ± 36.9	*	53.8 ± 124.4	*	46.5 ± 86.5	*	6.2 ± 8.3
Epitestosterone	542.4 ± 454	867.4 ± 118.8	228 ± 216	493.9 ± 767.6	61.2 ± 53.7	70.6 ± 76.4	62.2 ± 45.9	91.7 ± 101
Estradiol 3‐SO_4_	*	1241 ± 1277	*	1920 ± 4921	*	2477 ± 5472	*	750.0 ± 1187
Estriol‐3‐SO_4_	*	84.4 ± 110.4	*	80.2 ± 134	*	54.3 ± 106.8	*	19.2 ± 21.4
Estrone E_1_	*	912.4 ± 935.6	*	845.0 ± 936.2	*	266.3 ± 253.1	*	141.0 ± 89.3
Estrone‐SO_4_	*	9567 ± 7658	*	8278 ± 5472	*	2477.7 ± 5472	*	750 ± 1187
20α‐Hydroxy‐5α‐pregnan‐3‐one	84.2 ± 105	126.4 ± 437.7	299.9 ± 351.9	76.8 ± 266	89.4 ± 119.4	88.2 ± 194.5	83.8 ± 65.3	10.6 ± 35.1
11α‐Hydroxy‐4‐pregnene‐3	55.1 ± 69.2	532.9 ± 1061	45.9 ± 102.2	4649 ± 10 439	101.3 ± 148.1	792.4 ± 1332	85.4 ± 187.6	183.3 ± 493.4
11β‐Hydroxy‐4‐pregnene‐3	61 ± 99.7	1497 ± 2470	27.2 ± 42.7	1561 ± 1038	40.3 ± 79.4	652.2 ± 852.1	32.5 ± 60.4	702.9 ± 1197
21‐Hydroxypregnanolone	2227 ± 3439	89.3 ± 309.5	1917 ± 3477	2676 ± 9270	959.0 ± 1433	1161 ± 3471	608.3 ± 1502	774.2 ± 1963
17‐Hydroxypregnenolone	1119.5 ± 1518	2302 ± 4880	854.0 ± 2139	7033 ± 11 060	823.6 ± 1411	106 225 ± 224 348	139.1 ± 304.5	86 016 ± 91 591
20‐Hydroxypregnenolone	95 ± 189.5	6690 ± 12 184	367.2 ± 613.6	6628 ± 12 154	58.5 ± 107	13 782 ± 22 378	209.8 ± 347.3	6003 ± 7501
17‐Hydroxyprogesterone	3.4 ± 5.6	157.6 ± 161.9	5.2 ± 8.8	806.2 ± 1333	5.3 ± 7.9	512.5 ± 868.6	4.5 ± 8.4	437.1 ± 416.6
Pregnanolone	36 588 ± 19 943	410.9 ± 650.7	50 934 ± 37 193	6251 ± 14 273	16 391 ± 5148	978.3 ± 808.5	5923 ± 5148	446.6 ± 732.5
Pregnanediol	563.4 ± 935.3	2291 ± 4935	270.1 ± 532.6	1976 ± 2668	38.8 ± 74.4	850.1 ± 1376	0	32.9 ± 109
Pregnenolone	6972 ± 4038	1062 ± 1208	9653 ± 6976	4762 ± 11 195	5593 ± 3523	1879 ± 2558	4075 ± 3695	1152 ± 1712
Pregnenolone‐SO_4_	*	874.7 ± 695.4	*	929.5 ± 681.8	*	275.3 ± 342.3	*	142.1 ± 170.6
Progesterone	12.9 ± 17.3	5679 ± 10 110	2.4 ± 4.7	3198 ± 2521	5.4 ± 8.7	1903 ± 1629	3.1 ± 4.5	2041 ± 2175
Testosterone	9.6 ± 7	0	8.1 ± 9.3	8.5 ± 20.5	6.8 ± 6.2	11.6 ± 40.2	5.0 ± 4.9	6 ± 13.4

### 3.1 Serum and saliva neurosteroid concentrations

**FIGURE 1:**
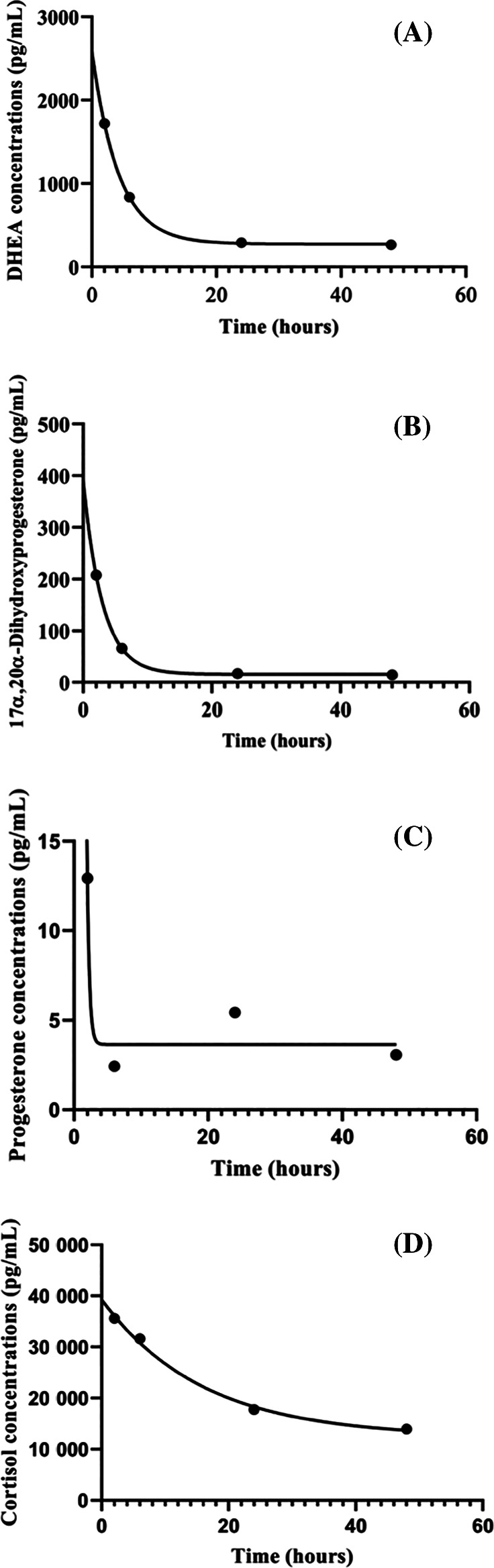
Decay curves of steroid compounds in serum over 48 hours in 12 calves. A, Decay curve for dehydroepiandrosterone (DHEA). *R* ^2^ = 0.99. Half‐life (95% CI) = 2.9 hours. B, Decay curve for 17α,20α‐dihydroxyprogesterone. *R* ^2^ = 0.99. Half‐life (95% CI) = 2.1 (1.3, 3.0) hours. C, Decay curve for progesterone. *R* ^2^ = 0.93. The half‐life for progesterone was 0.2 hours. However, 95% confidence interval, and rate constant were very wide, and undetermined suggesting that the decay curve was ambiguous and the half‐life estimate was not precise. D, Decay curve for cortisol. *R* ^2^ = 0.99. The half‐life for cortisol was 11 hours. However, 95% confidence interval for the half‐life and rate constant were wide, and undetermined suggesting that the decay curve was ambiguous and the half‐life estimate was not precise

All compounds were investigated in both, serum and saliva. However, 10 neurosteroid compounds in serum were best fitted with a 1‐phase exponential decay model. These compounds were DHEA, cortisol, cortexone, corticosterone, testosterone, 5α‐dihydroprogesterone, 21‐hydroxypregnanolone, 17α,20α‐dihydroxyprogesterone, pregnenolone, and pregnanediol. However, only DHEA and 17α,20α‐dihydroxyprogesterone were considered reference compounds because the 95% CIs for the half‐lives were determined. The serum half‐lives (95% CI) for DHEA (Figure 1A) and 17α,20α‐dihydroxyprogesterone (Figure 1B) were 2.9 (2.1, 4.3) and 2.1 (1.3, 3.0) hours, respectively. The 95% CI for half‐lives of the other 8 compounds were either very wide or undefined suggesting that the data points for these compounds did not unambiguously define the half‐lives, and therefore were not appropriate targets. Although serum progesterone and cortisol were not found to be target compounds in this study, they have been used as reference compounds in other species. Therefore, their 1‐phase decay curves were reported and depicted for comparison (Figure 1C,D). The half‐lives for progesterone and cortisol were 0.2 (undetermined, wide 95% CI) and 11 (undetermined, wide 95% CI) hours, respectively.

**FIGURE 2:**
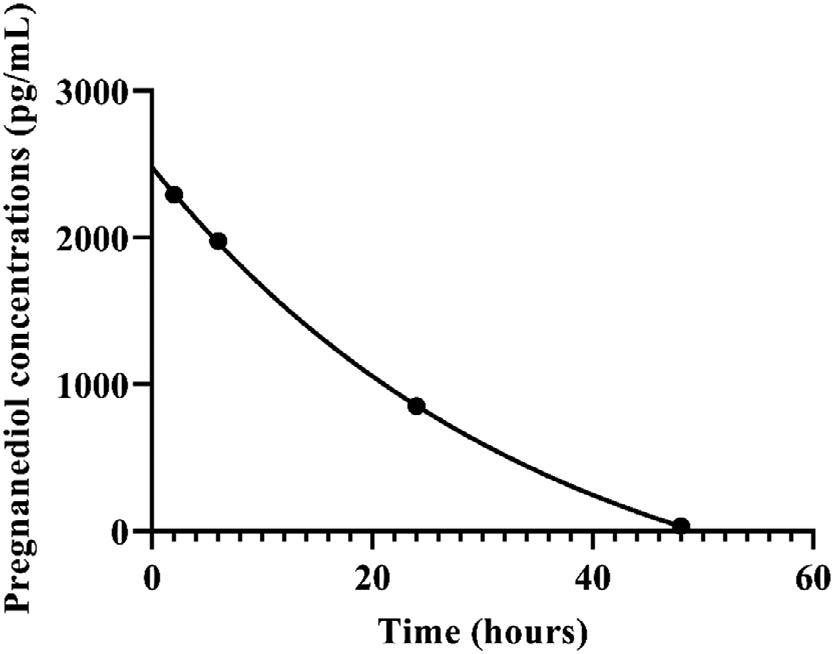
Decay curve for pregnanediol in saliva over 48 hours in 12 calves. *R* ^2^ = 0.99. Half‐life (95% CI) = 24.5 (14.2, 66.5) hours

Seven neurosteroid compounds in saliva including progesterone, cortisol, pregnanediol, epitestosterone, estrone‐E_1_, estrone‐SO_4_, and cholesterol‐3‐SO_4_ were best fitted by 1‐phase exponential decay models. However, pregnanediol was the only compound considered because the 95% CIs were calculated, whereas half‐lives of the other 6 compounds were either wide or undefined. The saliva half‐life (95% CI) for pregnanediol was 24.5 (14.2, 66.5) hours (Figure 2).

### 3.2 Comparison of serum and saliva neurosteroid concentrations

**FIGURE 3:**
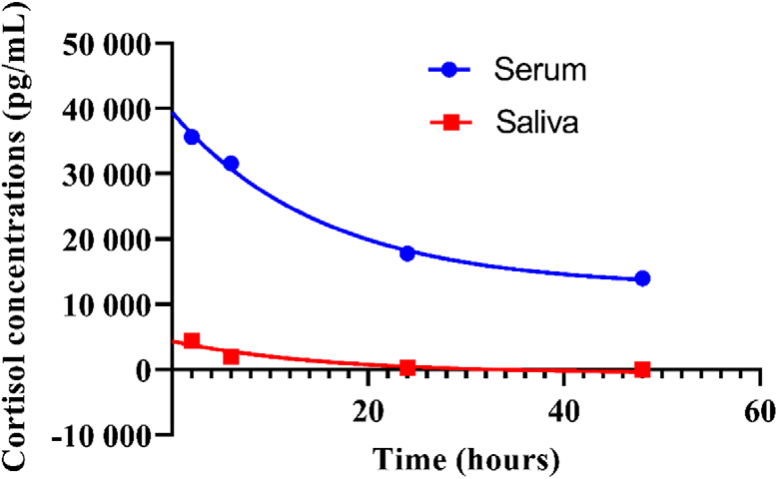
Comparison of decay curves for cortisol concentrations in serum and saliva in calves (N = 12). Rate constants between the curves are not different (0.06 for serum vs 0.21 for saliva; *P* = .29)

**FIGURE 4:**
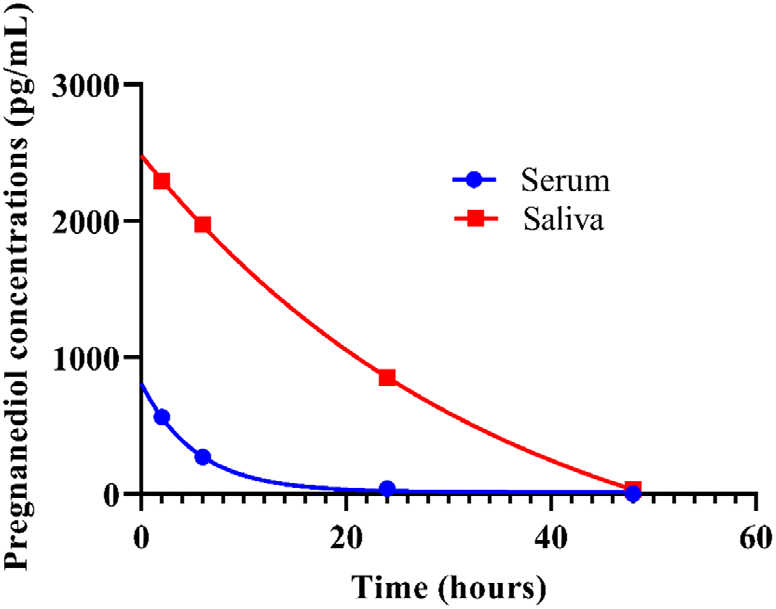
Comparison of decay curves for pregnanediol concentrations in serum and saliva in calves (N = 12). Rate constants between the curves are different (0.19 for serum vs 0.03 for saliva; *P* = .01)

Concentrations of cortisol and pregnanediol were compounds that were best fitted by 1‐phase exponential decay in both serum and saliva. The rate constants for the nonlinear regression models were not different (0.06 for serum vs 0.21 for saliva; *P* = .29) between serum and saliva cortisol concentrations (Figure 3). The rate constant for the nonlinear regression models were different (0.19 for serum vs 0.03 for saliva; *P* = .01) between serum and saliva for pregnanediol concentrations (Figure 4).

### 3.3 Calf and cow NS at birth

Comparison of cow to calf serum data at within 2 hours after birth yielded 3 group categories of NS. Concentrations for the NS were either higher, lower, or not different in calves compared to cows (Table 2).

**TABLE 2:** Comparison of serum steroid compounds concentrations (pg/mL) between cows and their respective calves at 2 hours after birth (N = 12). Concentrations are reported as median (range). Comparisons of concentrations were considered significant at *P* < .05

	Calves	Cows	*P* value
Steroid compounds higher in calves compared to cows			
Corticosterone	453.8 (0‐1502)	1.7 (0‐273.1)	.0008
Cortexone	85.9 (0‐147.9)	19.1 (0‐64.6)	.007
Cortisol	20 627 (5157‐139 445)	640 (0‐1224)	<.0001
Dehydroepiandrosterone (DHEA)	1331 (17.9‐8463)	55.4 (2.0‐154.7)	.0008
20α‐Dihydroprogesterone	192.7 (101.8‐870.9)	16.8 (4.3‐47.2)	<.0001
5β‐Dihydroprogesterone	67.8 (0‐191.8)	1.6 (0‐49.9)	.02
17α,20α‐Dihydroxyprogesterone	134.3 (35.4‐674.2)	27.2 (12.6‐104.8)	.001
Epitestosterone	406.5 (32.8‐1649)	117.0 (4.5‐184.4)	.003
Pregnanolone	32 734 (10 398‐59 004)	44.8 (0‐524.4)	<.0001
Pregnenolone	58 342 (1121‐15 599)	45.1 (0‐502.8)	<.0001
Steroid compounds lower in calves compared to cows			
Allopregnanolone	0	50.4 (16.6‐84.2)	<.0001
Androstenedione	0 (0‐18.4)	124.4 (13.0‐287.8)	<.0001
Progesterone	4.3 (0‐47.6)	99.9 (49.8‐137.5)	<.0001
Steroid compounds not different between calves and cows			
Aldosterone	0 (0‐4224)	18.4 (0‐271)	.44
Allodihydrotestosterone	61.1 (0‐169.6)	39.2 (1.0‐114.4)	.25
Cortexolone	0.08 (0‐39.3)	0.40 (0‐171.1)	.4
21‐Hydroxypregnanolone	0 (0‐9180)	3192 (0‐25 600)	.33
20α‐Hydroxy‐5α‐pregnan‐3‐one	42.8 (0‐331.1)	13.0 (0‐58)	.14
11α‐Hydroxy‐4‐pregnene‐3	12.2 (0‐171.6)	3.7 (0‐9.7)	.18
11β‐Hydroxy‐4‐pregnene‐3	0 (0‐298.3)	0 (0‐80.6)	.74
17‐Hydroxypregnenolone	385.3 (0‐4370)	19.3 (0‐75)	.31
20‐Hydroxypregnenolone	0 (0‐547.9)	0 (0‐94.8)	.21
5α‐Dihydroprogesterone	0 (0‐171)	0 (0‐76.6)	.56
17‐Hydroxyprogesterone	0 (0‐16.9)	1.6 (0‐14.6)	.25
Pregnanediol	0 (0‐2926)	13.9 (0‐234.9)	.61
Testosterone	9.5 (0‐23.7)	8.3 (0.8‐23.5)	.81

## 4 DISCUSSION

Based on our study, healthy neonatal Holstein heifers are born with high concentrations of steroid compounds with a variable decline over 48 hours after birth. However, 3 NS were found to have a consistent, rapid, and steady decline within the 48 hours of sampling. These NS were DHEA and 17α,20α‐dihydroxyprogesterone in serum, and pregnanediol in saliva. Dehydroepiandrosterone and 17α,20α‐dihydroxyprogesterone had the most precipitous decrease by 24 hours after birth, reaching their lowest concentrations that remained throughout the next 24 hours (48 hours after birth). Pregnanediol concentrations in saliva had a steady but less precipitous decline reaching low levels by 48 hours after birth. This rapid decline was because of the short half‐life of these NS, 2.9 and 2.1 hours in serum for DHEA and 17α,20α‐dihydroxyprogesterone, respectively; whereas 24.5 hours in saliva for pregnanediol. This rapid decline likely reflected a placental origin or termination of endogenous production.^11^ Similar findings occur in healthy neonatal foals in which steroid compounds such as progesterone, deoxycorticosterone, DHEA‐S, DHEA, and pregnenolone concentrations are elevated at birth and have a rapid decline within the first 48 hours of life with progesterone and pregnenolone having the most precipitous decrease by 24 hours after birth.^11^ Despite not following 1‐phase decay as other steroid compounds identified here as potential target compounds, serum progesterone (short half‐life of 0.2 hours) should be further investigated in a larger number of calves and beyond 48 hours after birth.

Upon comparison of steroid compounds concentrations in both serum and saliva in neonatal calves, 2 compounds (cortisol and pregnanediol) had the best fitted exponential decay within 48 hours after birth. Of these compounds, cortisol concentrations had a decay curve comparable between the 2 samples; whereas pregnanediol concentrations had a different decay curve between serum and saliva. Higher cortisol concentrations in plasma than in saliva occur in non‐neonatal calves.^29^ An explanation for this observation is that total cortisol (free and protein bound cortisol) is measured in plasma; whereas free cortisol which reflects concentrations in blood is measured in saliva.^29^ Furthermore, factors such as cortisol translocation from circulation to saliva and the rate of saliva production (eg, dilution effect) can affect cortisol concentrations in saliva.^29^ In this study, cortisol concentrations in serum became undetectable in saliva by 48 hours after birth. Based on the variability of results, saliva might not be the best sample to use in the investigation of steroid compounds in neonatal calves. Possible explanation for this diverse decay of NS in saliva could include its variable composition affected by electrolytes, IgG concentrations or other colostrum components (absorbed from the blood), milk or milk replacer composition fed to the calves, method of collection, and inability to accurately estimate half‐life of NS in saliva because of their wide confidence intervals.^30^ Colostrum or milk components in saliva might interfere with the extraction of steroid compounds by liquid chromatography‐mass spectrometry.^31^ Less variability in NS concentrations and decay was observed in serum samples. Furthermore, saliva collected from calves might contain particles such as bedding or hair follicles or any objects that calves might lick; thereby making sample processing difficult.

To assess if similar steroid compounds and concentrations were present in neonatal calves to those of their cows, serum samples were collected from both groups 2 hours after birth of their respective calves. Our results showed a wide variation in these compounds concentrations (Table 2). Neurosteroids and NAS concentrations that were significantly higher in calves than in cows included DHEA, cortisol, cortexone, corticosterone, 20α‐dihydroprogesterone, 17α,20α‐dihydroxyprogesterone, 5β‐dihydroprogesterone, epitestosterone, pregnanolone, and pregnenolone. A possible explanation for these higher concentrations in calves could represent endogenous production during fetal life; however contribution of concentrations from placental origin could not be excluded. DHEA and DHEA‐sulfate are secreted in high concentrations during fetal life in primates, mice, and humans.^32^, ^33^ These 2 compounds are dynamically regulated from before birth and have a key role in modulating early brain development, plasticity, and behavior during childhood.^33^ Therefore, it is possible that high DHEA concentrations at birth might reflect the importance of this compound in early neurodevelopment during fetal life and modulation of behavior after birth in neonatal calves. Parturition in cattle is initiated via the fetal pituitary‐adrenal axis.^34^ During late gestation, ACTH from the fetal pituitary gland stimulates the fetal adrenals to produce increased concentrations of cortisol.^34^ Fetal cortisol induces synthesis of placental 17α‐hydroxylase and aromatase, increased production of estrogen, and decreased production of progesterone in preparation for parturition.^35^, ^36^ Therefore, the increased cortisol concentrations in these neonatal calves also reflected endogenous production. Compounds found in significantly lower concentrations in calves included androstenedione, allopregananolone, and progesterone. Progesterone is needed to maintain pregnancy and assumed to be the reason for being higher in cows than in neonatal calves.^37^ Furthermore, through a series of enzymatic reactions allopregnanolone is derived from progesterone^2^; potentially also explaining why allopregnanolone is also higher in cows than in neonatal calves. Additionally, allopregnanolone increases during pregnancy and peaks in late gestation in rats and mares.^38^, ^39^, ^40^ The remaining steroid compounds studied here were not significantly different between calves and their cows and could have reflected a placental contribution. This was further supported by the observed decay in these compounds in subsequent time point collections in calves.

Increased NS and NAS concentrations occur in foals with NMS and sepsis.^17^, ^18^ It is postulated that the lack of transition of high NS and NAS concentrations reflecting those from fetal life to a rapid decline of concentrations after birth results in alterations of behavior and neurological function in foals with NMS. This failure to transition results in maladaptation to extrauterine life, displaying failure to thrive, and signs such as lack of bonding with the mare, not nursing, unawareness of the environment, obtundation, abnormal sleep, and in some cases seizures.^17^, ^18^, ^41^ Similarly, neonatal calves with NE display failure to thrive and inability to nurse.^23^ The prevalence of NE was reported to be 29% in 200 neonatal calves and with prompt proper supportive care, survival rate was 77.6%, similar to that in foals of 79.8%.^23^, ^42^ However, recovery time including regaining ability to nurse might take a few days which involves higher costs of hospitalization and nursing care.^23^ Identified risk factors for the development of NE in neonatal calves include dystocia with malpresentation of the calf in the birth canal.^23^ Given that over 33 million calves are born in the United States annually^43^; monitoring of calves with risk factors, early detection of NE, and proper medical intervention of affected calves are essential. Measurement of DHEA, 17α,20α‐dihydroxyprogesterone, and pregnanediol concentrations might aid in the early recognition of disease. Different NS and NAS have different effects that can vary from excitatory (eg, seizures) to inhibitory (eg, altered state of consciousness, sedation) and account for the displayed neurological signs.^1^, ^2^, ^5^, ^7^ Furthermore, knowing which steroid compounds are elevated might translate into potentially using reversal agents to counteract their effects. Future studies should consider serial measurement of DHEA and 17α,20α‐dihydroxyprogesterone in calves exposed to known risk factors for NE such as dystocia compared to normally delivered calves.

Limitations to this study included the low number of neonatal calves of exclusively heifer Holstein calves. This is important because male calves and breeds used for beef (96%) vs milk (4%) production were overrepresented in the NE group in 1 study.^23^ However, this observation might have represented management practices and overall hospital population.^23^ Further studies including larger numbers of animals, breeds, production type, and of both sexes are warranted. Other limitation included the lack of studying NS and NAS concentrations in other body fluids such as urine and cerebrospinal fluid. For instance, DHEA concentrations have been studied in urine from older calves after oral and intramuscular administration of DHEA.^44^ However, naturally occurring DHEA concentrations have not been investigated in neonatal calves. Despite these limitations, this is the first study to investigate NS and NAS concentrations in healthy neonatal calves. In our study, NS and NAS concentrations were only determined up to 48 hours after birth in contrast to studies in foals where concentrations were determined until 7 days of age.^11^ Determination of steroid compounds concentrations beyond 48 hours might affect the decay curves characteristics because of additional time point measurements. Consequently, additional reference compounds might be considered as potential targets when measured beyond 48 hours. Therefore, our study results cannot entirely exclude potential targets including progesterone that have been identified in foals.

In conclusion, DHEA and 17α,20α‐dihydroxyprogesterone were found to rapidly decline within 24 hours of life and remained at low concentrations in serum throughout 48 hours after birth in healthy neonatal Holstein heifers. Overall, less variability in NS and NAS concentrations and decay over time were observed in serum compared to saliva samples. Based upon comparison of NS and NAS concentrations in serum from both cows and their respective calves at 2 hours after birth, high concentrations of DHEA and 17α,20α‐dihydroxyprogesterone in serum of neonatal calves likely reflected endogenous production. The steady decline with very low concentrations of pregnanediol in saliva by 48 hours after birth in neonatal calves and no significant difference of this compound in serum from both cows and their respective calves was more likely reflecting a placental origin. DHEA and 17α,20α‐dihydroxyprogesterone in serum, and pregnanediol in saliva could be used as potential markers of disease in neonatal heifer calves with unexplained failure to thrive or encephalopathy of unknown etiology. However, because of the wide 95% CI of the half‐life, pregnanediol in saliva might not be an ideal marker.

## CONFLICTS OF INTEREST DECLARATION

Munashe Chigerwe serves as Associate Editor for the *Journal of Veterinary Internal Medicine*. He was not involved in the review of this manuscript. No other authors declare a conflict of interest.

## OFF‐LABEL ANTIMICROBIAL DECLARATION

Authors declare no off‐label use of antimicrobials.

## INSTITUTIONAL ANIMAL CARE AND USE COMMITTEE (IACUC) OR OTHER APPROVAL DECLARATION

The study was reviewed and approved by UCD‐IACUC as described to collect blood and saliva samples from neonatal calves at 2, 6, 12, 24, and 48 hours of age. The study was non‐invasive and not terminal. No animals were harmed.

## HUMAN ETHICS APPROVAL DECLARATION

Authors declare human ethics approval was not needed for this study.
